# Conservative Sex and the Benefits of Transformation in *Streptococcus pneumoniae*

**DOI:** 10.1371/journal.ppat.1003758

**Published:** 2013-11-14

**Authors:** Daniel J. P. Engelmoer, Ian Donaldson, Daniel E. Rozen

**Affiliations:** 1 Faculty of Life Science, University of Manchester, Manchester, United Kingdom; 2 Department of Animal Ecology, Faculty of Earth and Life Sciences, Vrije Universiteit Amsterdam, Amsterdam, The Netherlands; 3 Institute of Biology Leiden, Leiden University, Leiden, The Netherlands; State University of New York at Stony Brook, United States of America

## Abstract

Natural transformation has significant effects on bacterial genome evolution, but the evolutionary factors maintaining this mode of bacterial sex remain uncertain. Transformation is hypothesized to have both positive and negative evolutionary effects on bacteria. It can facilitate adaptation by combining beneficial mutations into a single individual, or reduce the mutational load by exposing deleterious alleles to natural selection. Alternatively, it may expose transformed cells to damaged or otherwise mutated environmental DNA and is energetically expensive. Here, we examine the long-term effects of transformation in the naturally competent species *Streptococcus pneumoniae* by evolving populations of wild-type and competence-deficient strains in chemostats for 1000 generations. Half of these populations were exposed to periodic mild stress to examine context-dependent benefits of transformation. We find that competence reduces fitness gain under benign conditions; however, these costs are reduced in the presence of periodic stress. Using whole genome re-sequencing, we show that competent populations fix fewer new mutations and that competence prevents the emergence of mutators. Our results show that during evolution in benign conditions competence helps maintain genome stability but is evolutionary costly; however, during periods of stress this same conservativism enables cells to retain fitness in the face of new mutations, showing for the first time that the benefits of transformation are context dependent.

## Author Summary

Transformation of environmental DNA can provide bacteria with a means to adapt quickly to a changing environment. While this can benefit microbes by facilitating the spread of antibiotic resistance, it can also be harmful if it causes the loss of beneficial alleles from a population. Therefore, it is unclear what evolutionary factors enable transformation to persist in bacterial populations. We used the naturally transformable opportunistic pathogen *Streptococcus pneumoniae* to investigate the long-term benefits of transformation. We compared the fitness of laboratory populations of *S. pneumoniae* after 1000 generations of evolution. Half of these populations were naturally transformable (competent) while the other half was deficient for this function. At the same time, half of the evolving populations were periodically exposed to short periods of mild stress. We find that competence reduces the average fitness gain of evolving populations, but this cost is mitigated in populations facing mild stress. Using whole genome sequencing, we discovered that functional competence reduces the total number of fixed mutations and prevents hyper-mutable cells from increasing in frequency. Our results suggest that competence in *S. pneumoniae* is a conservative process acting to preserve alleles, rather than an innovative one that persists because it recombines beneficial mutations.

## Introduction

Natural transformation is an important cause of genome evolution in bacteria, but the evolutionary factors maintaining natural transformation, or competence, in bacteria remain uncertain [1], [2], [3], [4]. Transformation is widely believed to have evolved to facilitate adaptation, especially in a clinical context where transformation occurs at a high rate and may allow pathogens to evade antibiotics or immune surveillance [2], [5], [6], [7]. However, transformation can both be beneficial and costly to bacterial cells. It can speed up adaptation by combining non-antagonistically epistatic beneficial mutations into a single individual [8], [9], [10], [11], similar to the Fisher-Muller effect in Eukaryotes. It can also reduce the mutation load by combining deleterious alleles into a common background, which more efficiently exposes these mutations to natural selection [12], [13], [14], [15]. Similarly, transformation can eliminate deleterious alleles if these are replaced by transformed DNA with the wild-type sequence [12]. Such a function has been inferred in naturally transforming *Neisseria*, where high rates of transformation with ‘self-DNA’ leads to conservation of core regions of the genome [16]. Alternatively, transformation can impose significant fitness costs because it is energetically expensive and bacterial cells may incorporate damaged or mutated environmental DNA that reduces bacterial fitness [12]. Thus, although the mechanisms that regulate bacterial transformation are well understood, the evolutionary factors that maintain this process are not.

Experiments designed to quantify the evolutionary effects of bacterial transformation have been equivocal. While Baltrus et al. [17] showed that transformable strains of *Helicobactor pylori* evolved more rapidly than non-transformable strains, Bacher et al. [18] found the reverse in *Acinetobacter baylyi*. Indeed, wild-type strains in this experiment evolved lower rates of transformation during laboratory culture. Although these experimental differences may have been caused by details particular to the species investigated, they may have also been caused by experimental approaches that inadvertently exposed different benefits or costs of competence. For example, studying yeast, *Saccharomyces cerivisiae*, Gray and Goddard [19] showed that sex only increased adaptation in a stressful environment, and that this was especially pronounced in populations of cells with a high mutation rate. Similar studies designed to partition the effects of these experimental factors have thus far not been attempted using bacteria.

Here, we use an experimental evolution approach to examine the long-term effects of transformation on the naturally competent opportunistic pathogen *Streptococcus pneumoniae*. Replicate cultures of a competent strain and an isogenic mutant unable to become competent were evolved for 1000 generations in two different chemostat environments. Half of the populations were evolved in a constant benign environment, while the other half was exposed to twice-weekly pulses of a sub-minimum inhibitory concentration (MIC) of kanamycin. This treatment was used to examine context-dependent effects of transformation. Although the concentration of kanamycin we used did not influence the growth rate of cells in our experimental populations (Figure S1), it is sufficient to induce cellular stress and also induce natural competence [20]. In brief, we found that competent cells evolved in a benign environment increased in fitness less than non-competent cells; however, this cost of competence was alleviated when cells evolved in an environment where they were challenged with periodic stress. Using whole genome sequencing of evolved isolates, we show that non-competent populations fixed more mutations than competent ones and are furthermore were more likely to evolve to become mutators. Our results provide direct experimental evidence that the effects of transformation are context dependent. In benign conditions the effects of transformation are conservative and evolutionarily costly; however, this same conservativism benefits cells living in stressful environments.

## Results

### Conditional effects of competence

To quantify the effects of competence on the evolution of *S. pneumoniae* we evolved replicate populations of a competent wild-type and an isogenic non-competent mutant in chemostats for 1000 generations. We introduced periodic stress in half of the evolving populations by applying sub-MIC concentrations of kanamycin to chemostat vessels twice each week. Kanamycin was added as a single injection to the chemostat sampling port in order to achieve a final concentration of 5 ug/mL, which is approximately 20-fold below the MIC for this strain. At the chemostat flow rates used, the kanamycin was eliminated within 7 hours. For the majority of time, these populations therefore experienced the same environment as the unstressed populations. At the start of this experiment, all ancestral populations exhibited pronounced oscillations of several orders of magnitude in cell density (between 10^9^/ml and 10^5^/ml), as previously described with this species [21]. However, within 500 generations these fluctuations were uniformly lost [22], and all populations retained a stable density of 10^9^ cells/ml. As a consequence of this change, which made it impossible to directly compete evolved and ancestral cells, fitness differences between treatments after experimental evolution were estimated using pair-wise competition assays between differentially marked terminal populations [23]. This allowed us to directly estimate fitness differences between evolved strains, and to quantify the relative fitness of competent and non-competent populations under benign and periodically stressed conditions.

**Figure 1:**
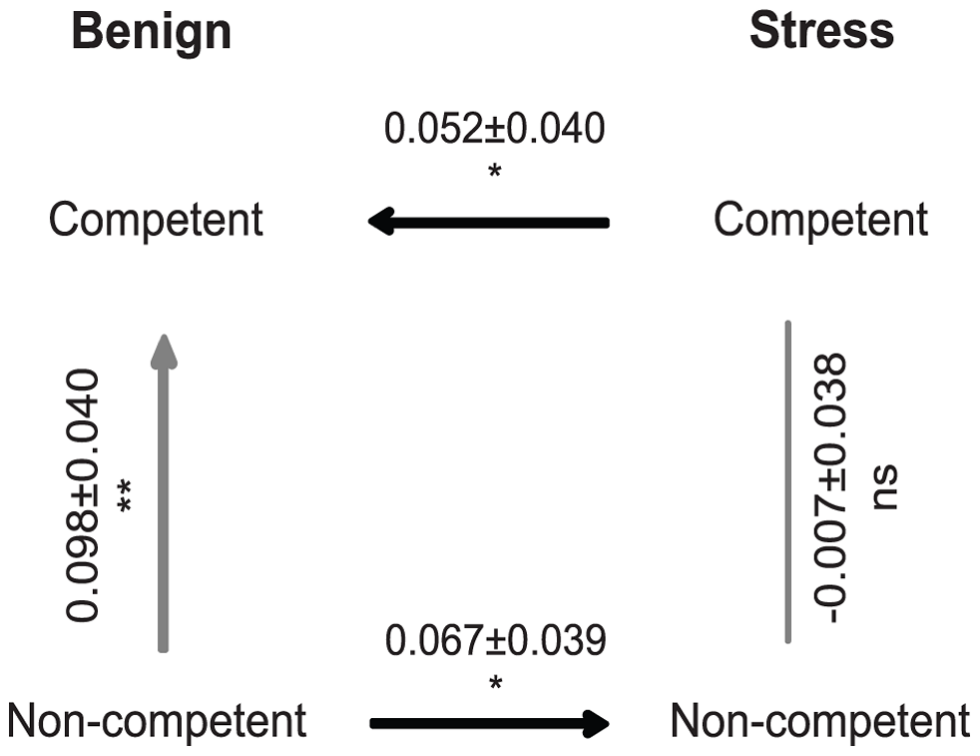
Fitness differences between experimental treatments estimated from direct competition assays between evolved populations. Values represent the mean selection rate constant/hour (± standard error). Ns: non-significant; *: P<0.05; **: P<0.01 compared to the null expectation of 0 (i.e. equal fitness).

In contrast to the expectation that competence accelerates adaptation, we found instead that evolved competent populations of *S. pneumoniae* were significantly less fit than non-competent populations (Fig. 1, restricted maximum likelihood (REML) mixed model compared to 0: t = −2.22; p<0.001). This evolutionary cost of competence corresponds to a fitness difference between evolved populations of ∼0.098/hr, which implies that under benign conditions the effects of competence on adaptation are significantly negative. By contrast, the relative fitness of competent and non-competent populations evolved in the presence of periodic stress was indistinguishable (Fig. 1, REML mixed model compared to 0: t = −0.18; p = 0.741) indicating that these conditions significantly offset the evolutionary costs of competence (REML mixed model: F = 2.077; p = 0.017).

Exposure to periodic stress could offset the costs of competence by increasing adaptation of competent cells and/or by reducing the rate of adaptation of non-competent cells. The results from competition assays support both explanations. When we estimated the relative fitness of stressed versus unstressed competent cells we found that stressed populations were significantly more fit (∼0.052/hr) than populations evolved in an unstressed environment (Fig. 1, REML mixed model compared to 0: t = 1.27; one-tailed p = 0.045). By contrast, we found the reverse relationship for non-competent cells, and of roughly equal magnitude; stressed non-competent populations were significantly less fit (∼0.067/hr) than populations evolved in an unstressed environment (Fig. 1, REML mixed model compared to 0: t = −1.67; one-tailed p = 0.012). The sum of these two effects on the rate of adaptation, i.e. the benefit for stressed competent populations and the cost for stressed non-competent populations, is similar to the ∼0.098/hr cost for competent cells noted above (Fig. 1). The benefits to competence of stress therefore arise in two ways: by increasing adaptation of competent cells and by enabling competent cells to avoid fitness reductions attributable to exposure to stress.

### Genomic effects of competence

**Figure 2:**
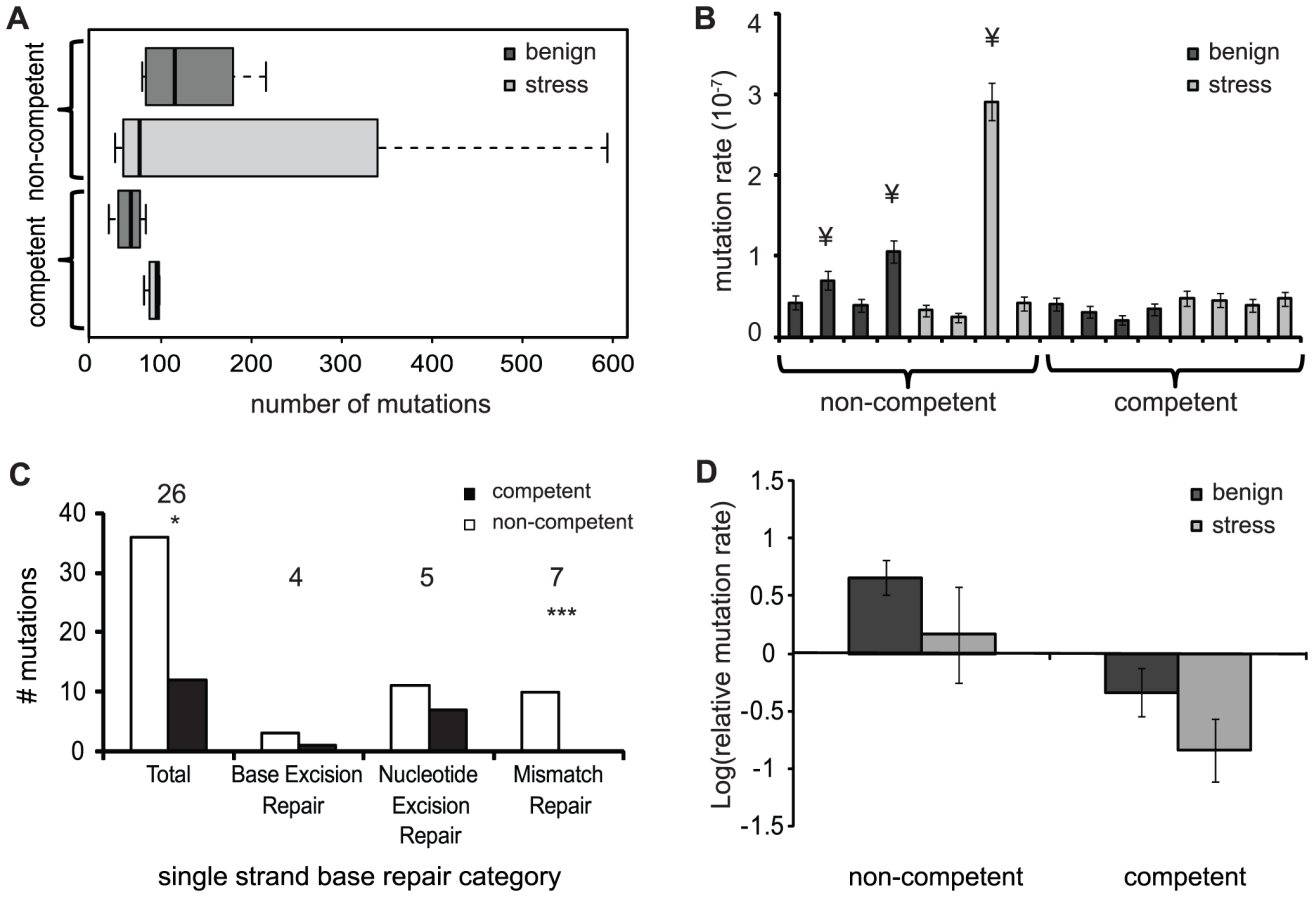
Number of mutations and mutation rates of evolved populations. Dark grey are populations evolved in the benign environment and light grey are the populations evolved in the periodic stress environment. A) The average number of mutations found per evolved population in each treatment. B) Mutation rate for each evolved population calculated from the total number of mutations. ¥ indicates populations with a mutation rate of 2–10-fold above average. Error bars are 95% confidence intervals C) Mutations found in genes for DNA repair for non-competent (white) and competent (black) populations. Numbers above each category indicate the number of mutated genes in that category. * Indicates significant difference between competent and non-competent populations. D) Mean of log-transformed relative mutation rates of the evolved lines compared to the ancestor. Error bars are SE of the mean.

To analyze the influence of competence and stress on genome evolution during experimental evolution we obtained the complete genome sequences of evolved clones from each of the 16 populations together with their respective ancestors. Competence could potentially alter mutation fixation in two ways, both caused by the fact that competence unites mutations from separate cells into a common genetic background. First, if recombined mutations are beneficial either alone or in combination, competence could increase the fixation rate because recombinant cells would be predicted to increase in frequency. Second, if recombined mutations are deleterious, alone or in combination, competence could reduce the fixation rate because recombinant cells would be exposed to natural selection and eliminated from the experimental population. A similar reduction in fixation rate would be anticipated if competence replaces new mutations in the host genome with donor DNA containing the wild-type allele, thereby “correcting” mutations. We furthermore predict that sub-MIC antibiotic stress will have a general increase on mutation fixation, owing to potentially mutagenic effects of kanamycin [24]. Sequencing of evolved genomes identified a total of 421 synonymous mutations and 1282 non-synonymous mutations across all evolved lines. Substitutions were not evenly distributed across treatments and populations, however. Consistent with the second possibility outlined above, we found that the total number of mutations in competent populations was significantly lower than in non-competent populations (Fig. 2A; GLM: z = −9.344, df = 1 p<0.0001). Moreover, the total number of mutations was significantly higher in populations experiencing periodic stress for both competent and non-competent populations (Fig. 2A; GLM: z = 7.379, df = 1 p<0.0001).

We next determined the mutation rate of each population based on the total number of mutations. This method assumes that the mutation rate was constant over the period of evolution and that there are no back mutations. We found that three of eight non-competent populations substituted between two and eight-fold more mutations than the other populations (Fig. 2B), suggesting that these lineages had evolved to become genetic mutators. However, because this estimate assumes a constant mutation rate during the 1000 generations of experimental evolution, rather than a changing rate due to the fixation of mutator alleles, mutators emerging at the end of experimental evolution may not be identified. To address this limitation, we first searched for mutations in genes diagnostic for bacterial mutators (e.g. DNA-repair genes *polC*, *dnaQ*, *rpoABC* and *mutL* [25], see Table S1 for a comprehensive list). We focused here on non-synonymous mutations because these are more likely to cause functional defects in the relevant genes. Next we determined the mutation rate of all evolved lineages relative to their respective ancestor using a phenotypic assay designed to detect the frequency of spontaneous mutants to resistance to either rifampicin or streptomycin [26]. Using the first approach, we detected significantly more non-synonymous mutations in DNA repair genes in non-competent than in competent populations (Fig. 2C: GLM one-tailed: χ^2^ = 162.00, df = 1, p = 0.0478), an effect that is most pronounced in genes for mismatch repair (Fig. 2B; GLM: Mismatch repair: χ^2^ = 9.875, df = 1, p = 0.001). Next, using a phenotypic assay, we found that the mutation frequency of non-competent lineages was significantly higher than competent populations (Fig. 2D; Two-way ANOVA: F_1,11_ = 10.189; p = 0.009), although the mutation frequency of ancestral strains were indistinguishable (t-test with unequal variances: t = 1.1336, df = 7.112, p = 0.2937). In contrast to re-sequencing results, these assays found no overall effect of stress on the mutation frequency (Fig. 2D; Two-way ANOVA: F_1,11_ = 2.699; p = 0.129), nor an interaction between stress and competence (Fig. 2C; Two-way ANOVA: F_1,11_ = 0.0003; p = 0.9876). Thus both at the genetic and phenotypic levels, our data support a model where competence reduces mutation fixation and limits the emergence of mutator phenotypes, but this conservatism comes possibly at the expense of reduced adaptation under benign growth conditions.

## Discussion

Transformation can dramatically benefit *S. pneumoniae* by facilitating the evolution of drug resistance and the emergence of novel modes of virulence [27], [28], [29]. However, these benefits in pathogenic bacterial lineages under strong antibiotic selection tell only part of the story, and may not reflect the effects of transformation more broadly. Using an experimental evolution approach, we found that competence benefited cells by reducing the mutation load and limiting the emergence of mutators (Fig. 2). Additionally, competent populations reached higher fitness when evolving in the presence of periodic stress; equally, exposure to periodic stress decreased the rate of evolution of non-competent populations (Fig. 1). Although we applied an extremely mild stress in our experiment (Figure S1), it is notable that the kanamycin concentration we used is sufficient to induce competence in wild-type strains [20]. It is therefore possible that benefits to competence in populations that experienced drug-stress was the result of increased recombination, which could have off-set the cost of transformation in a benign environment by slightly increasing their rate of adaptation. By contrast, non-competent cells exposed to kanamycin may face greater costs because kanamycin causes an inability to repair ribosomal decoding errors, which can subsequently lead to DNA damage and increase the mutation rate [24]. These stress-dependent benefits of competence may be particularly important in the human nasopharynx, where *S. pneumoniae* is exposed to unpredictable and severe stress from drug exposure, immune surveillance and from coexisting bacterial competitors.

Transformation is predicted to benefit bacterial species with high mutation rates by reducing their mutation load [12]. Using complete genome sequences, we estimate that the average mutation rate in *S. pneumoniae* is ∼3.8×10^−8^ per bp per generation. This corresponds to U = 0.08 mutations per genome per generation, or about 200-fold higher than *Escherichia coli* [30], yet similar to other naturally transformable opportunistic pathogens, such as *H. pylori* and *H. influenzae* [30], [31], [32], [33]. Despite these high rates of mutation we were surprised to find that some of the non-competent strains evolved even higher rates of mutation than their ancestor during this long-term experiment (Figs. 2A and 2B). These genotypic results were confirmed phenotypically (Fig. 2D), and suggest that our genomic data underestimates the difference in fixation rates in competent and non-competent populations. Although we are uncertain what caused the difference in mutation rates between competent and non-competent lineages to arise, one strong possibility is that transformation separates mutator alleles from the mutations they cause. Thus while mutations in DNA repair genes leading to mutators may arise equally in both competent and non-competent cells, they are lost before they become common in competent lineages [34]. Accordingly, competent lineages fix fewer mutations overall. Under benign conditions this may limit adaptation while causing minimal harm to non-competent populations. However, non-competent cells suffer to a greater degree when faced with stress, because they cannot revert to a less loaded state, and because stress may exacerbate the negative fitness effects of new mutations [35], [36]. In a similar recent study with the yeast *Saccharomyces cerevisiae* sex neutralised the deleterious effects of hyper mutation on the rate of adaptation [19]. The neutralisation of potentially deleterious mutations, e.g. those that lead to hyper mutation, is an example of how transformation can function to conserve genome integrity. Similar effects are inferred in the naturally transformable bacterial genus *Neisseria* where the number of species-specific DNA uptake sequences (i.e. small sequence tags that identify that a DNA fragment is derived from a particular species) are more frequent in the core genome. This indicates that these core genes are often the target of ‘selfing’ events, by which. transformation stabilizes the integrity of key genes [16]. Although *S. pneumoniae* is much more promiscuous than *Neisseria* when it comes to environmental DNA choice, the mechanism that induces competence is assumed to lead to the uptake of DNA released from lysed cells of the same and/or very closely related species [37], which suggests that the conservative benefits we observe from competence in our study may extend more broadly to pneumococci in the natural environment.

Bacteria in nature face unpredictable patterns of stress and mutation. Our results suggest that these conditions, together with an intrinsically high mutation rate, favour the maintenance of transformation while infrequent stress may facilitate its loss. Notably, surveys of naturally competent species such as *H. influenzae* and *B. subtilis* have revealed that transformation rates among clones within species can vary by over 6 orders of magnitude [38], [39], [40]. Similar variation exists in *S. pneumoniae* [41], [42], [43], indicating that competence is readily gained and lost in this species. In summary, we conclude that competence in *S. pneumoniae* is a conservative process acting to preserve alleles, rather than an innovative one that persists because of benefits it provides by recombining beneficial mutations.

## Materials and Methods

### Strains, culture conditions and chemostats

Strains used in this study were derived from Rx1 and its isogenic non-competent derivative FP5, which is unable to secrete the competence stimulating peptide, CSP [42]. Spontaneous rifampicin or streptomycin resistant mutants were isolated from each strain, and then four independent colonies of each type were further sub-cloned and stored at −80°C. These four independent ancestors were selected based on similar growth rates (Figure S2) and mutation rates (mean rates ±95% CI Non-competent: 1.84*10^−7^±1.56*10^−7^, competent: 8.41*10^−8^±7.51*10^−8^; t-test with unequal variances: t = 1.1336, df = 7.112, p = 0.2937). These 16 total clones (2 strains×2 drug resistance types×4 replicates) represented the ancestral populations for experimental evolution. Cultures were grown in ¼ CTM pH 7.8 (Complete Transformation Medium), which per litre consists of: 7.5 g Tryptic Soy Broth; 0.25 g yeast extract; 6 g NaCl at pH 7.8. This environment supported high levels of transformation (Figure S3). Blood agar plates (Tryptic Soy Agar (TSA)+3% horse blood), supplemented, where necessary, with either 4 µg/mL rifampicin or 100 µg/mL streptomycin, were used to enumerate cell density within chemostats and for colony counting during competition assays.

Experimental evolution and competition assays were performed in custom-made chemostats with a 25 mL working volume and a flow rate of 4 mL/hr, whilst maintained at 37°C [29]. Chemostat cultures were inoculated and maintained as described previously [21], and sampled every 50 generations of growth. Samples were stored at −80°C as freezer stocks in ¼ CTM pH 7.8+25% v/v glycerol at an OD_600_ of 0.20, corresponding to a density of 2×10^8^ cells mL^−1^.

### Long-term evolution experiment

Sixteen chemostat populations were inoculated with independently picked clones from the original antibiotic resistant strain to generate 4 replicates each of a 2*2 treatment design pairing competence and stress. The replicates in each treatment were equally split between the two differently marked versions of Rx1 (competent strain) and Fp5 (non-competent strain). Half of the populations were exposed twice a week to low doses of kanamycin introduced directly into the chemostat to simulate short periods of stress. Kanamycin concentrations were 5 µg/mL upon introduction, but declined with the normal outflow rate of the chemostat. This concentration of kanamycin had no effect on the growth rate of cells (Figure S1), but is sufficient to cause ribosomal decoding errors during protein production, which promotes the induction of competence [20], [44]. For simplicity, this treatment is referred to as “periodic stress” and the basal treatment as “benign”. Each strain was evolved independently, thereby avoiding potential effects of cross-induction of competence or competence-induced cell-lysis [45], [46]. Every week, after approximately 50 generations, a 1 mL sample was taken from each population and tested for the presence of the correct marker and absence of the opposite marker. Contaminated populations were restarted with 50 µL of the previous sampled uncontaminated time point. Populations were maintained for 20 weeks, which corresponds to about 1,000 generations.

### Fitness assays

Fitness was determined by comparing the change in relative densities of two reciprocally marked evolved populations in a chemostat in mixed culture over a 32-hour span. This time period was chosen because it is within the period that the periodically stressed populations spend in the benign environment between doses of kanamycin. Competition assays were initiated by inoculating chemostats with equal densities of each competitor. Chemostats were sampled immediately and then again after 32 hours to determine the relative densities of each competitor. The Malthusian parameters per hour were then calculated for each strain based on the density of each strain at the start and end of the competition, as described previously [47]. The selection rate constant was then calculated as the difference between Malthusian parameters as described previously [23]. First, we tested for a significant fitness difference between competitors for each treatment by comparing a restricted maximum likelihood mixed model against an intercept of zero, corresponding to equal fitness. In the mixed model, replicate fitness assays of competitor combinations were nested as a random effect within the fixed effect of treatments (absence/presence periodic stress and absence/presence competence). Second, we used the restricted maximum likelihood (REML) mixed model, again with replicate fitness assays as a random factor within treatments, to test for fitness differences between treatments (periodic stress or competence as a fixed factor). All analyses were done in R with package LME4. P-values were estimated by MCMC simulation with 10,000-fold replication using the p.vals command from the languageR package.

### DNA isolation and sequencing

Clonal isolates from each of the 16 evolved populations as well as all four ancestral strains were sequenced using the SOLiD4 platform at the University of Manchester genomics facility. Genomic DNA was obtained using phenol-chloroform isolation and ethanol precipitation [48]. SOLiD data were normalised to an equal number of reads (8,315,863 per strain) for each sample using a custom perl script, that randomly sampled the reads from the original dataset (getRandomTags_Index_fastq.pl) developed by I. Donaldson. The normalisation equalized the size of the datasets to the strain with the lowest number of reads thereby normalising the quality of the consensus sequences. The normalised reads were then mapped against the fully sequenced reference strain *S. pneumoniae* R6 (genbank accession: NC_003098 = AE007317, an easily accessible version of the genome database can be found at http://www.streppneumoniae.com) using BFAST (0.6.4e) using default colour space methodology giving an average coverage depth of 151-fold. Mapped reads were then locally realigned around INDELs using SRMA (0.1.15). SNPs and small INDELs were then determined from the resulting BAM-files using the Geneious package (Geneious 5.4, Auckland, New Zealand; [49]. A SNP or INDEL was called when the change to the reference was supported in 60% of the reads with at least a coverage depth of 20 reads using the variant calling tool in Geneious 5.4 to minimise false positives and negative SNP calls. The 60% support is slightly less stringent than previous studies, but this is compensated by the high average coverage depth of 151 (±SE 2.53) reads/base [50], [51]. Subsequently, variant tables extracted from Geneious were used in the Galaxy online tool set [52], [53] to identify mutations for each evolved clone compared to its ancestor. Parallel changes were then double checked by hand in the UCSC microbial genome browser [54] to eliminate false positives. The resulting mutation tables were used for further analysis.

### Mutations and mutation rate

To determine the effect of periodic stress and competence the total numbers of mutations were compared in a GLM model with a Poisson distribution using R. Subsequently, two different methods were used to determine the mutation rate of evolved populations. First, the per base per generation mutation rate was calculated from genome data from the total number of mutations (See Table S2 for mutation rates based on coding mutations and synonymous mutations only); 95% confidence intervals for these mutation rates were determined in R using a Poisson distribution. This analysis assumes that the number of mutations is relatively small, that there are no back mutations, and that the mutation rate was constant over the period of evolution.

Second, the mutation frequency of terminal lineages (i.e. the number of spontaneous mutants with either rifampicin or streptomycin resistance/total population density) was determined following the methods in [26] to estimate the mutation rate of terminal populations isolated after their final generation of experimental evolution. Each strain and its corresponding ancestor was grown overnight at 37°C+5% CO_2_. Cells were then washed and concentrated by centrifugation and re-suspended in 100 µL 0.8% NaCl. 10 µL spots at several different dilutions (five spots per dilution) were plated onto blood agar plates supplemented with either 4 µg/mL rifampicin or 100 µg/mL streptomycin, whilst total cell densities were determined on unsupplemented plates. Mutation frequency was estimated as the ratio of the number of mutants to the total population size. Assays were performed in triplicate for each genotype, and the relative mutation frequency was determined as the ratio of the mutation frequency of each evolved lineage to its corresponding ancestor. Mean relative mutation frequencies were log-transformed before analysis using a two-way ANOVA.

Finally, parallel changes in DNA repair genes were examined at the level of each gene and functional group, as determined from the KEGG classifications for *S. pneumoniae* R6 (http://www.genome.jp/dbget-bin/www_bget?gn:T00060). The table of non-synonymous SNPs was used to create a SNP-by-gene table by scoring presence/absence of at least one SNP in a given gene for each strain (see Table S1 for a detailed summary of the genes involved and the amino acid changes found per evolved line). Functional group associations were created from the total SNP-by-gene table by summarising presence and absence of SNPs for genes associated with a functional group according to the KEGG-database. Generalised linear models were used to test for differences between treatments for parallel non-synonymous mutations in functional groups.

## Supporting Information

Figure S1 **Growth rate of ancestors exposed to different concentrations of kanamycin.** Growth rates were estimated at different concentrations of kanamycin for the four ancestors in a 96-well plate using an automated plate reader (n = 3). OD_600_ in CTM pH 7.8 was measured every 5 minutes for 24 hours at 37°C with continuous shaking. Raw OD values were normalised to a blank well and Ln transformed before analysis. Growth rates declined with increasing concentration of kanamycin (Figure S1).
(DOCX)

Figure S2 **Growth rates of the four ancestors.** Growth rates were estimated within a 96-well plate using an automated plate reader (n = 5). Cultures were started with 3*10^5^ cells in 200 µL of CTM pH 7.8. The OD_600_ was measured every 5 minutes for 24 hours at 37°C with continuous shaking. Raw OD values were normalised to a blank well and Ln transformed before analysis. Then the steepest slope over a 35 minute period was determined. The growth rates were analysed with a one-way ANOVA using R (Figure S2).
(DOCX)

Figure S3 **Natural transformation in the chemostat environment.** Populations containing mixes of reciprocally marked (rifampicin or streptomycin resistance) ancestors were tested for the frequency of cells with both markers to test for natural transformation in the chemostat environment. Double marked cells that were found in chemostats containing a mix of the non-competent FP5 ancestor would be the result of mutation, while those found in the chemostats with a mix of the competent Rx1 ancestor would be the result of both mutation and recombination. If the rate at which double mutants occur in the mix of Rx1 is higher than the mix of FP5 then this excess will be the result of transformation. To test this, chemostats containing ¼ CTM pH 7.8 were inoculated with either a 1∶1 mix (n = 3 for each mix) of streptomycin and rifampicin resistant FP5 (non-competent) or Rx1 (competent). After 24 hours of growth at 37°C, the population density and the number of double marked cells was determined on blood agar plates, which were supplemented with 100 µg/mL streptomycin and 4 µg/mL rifampicin where necessary. Since the population sizes were similar after 24 hours, the number of double marked cells in the total population was compared between ancestor types in R using a GLM model with a Poisson distribution (Figure S3).
(DOCX)

Table S1 **Table of all mutations across DNA repair genes for all evolved populations.**
(DOCX)

Table S2 **Mutation rates calculated from mutations in coding sites and synonymous sites.**
(DOCX)
