# Supplementary material for: Conservative Sex and the Benefits of Transformation in Streptococcus pneumoniae
Source: PLoS Pathog. 2013 Nov 14;9(11):e1003758. doi: 10.1371/journal.ppat.1003758 (PMC3828180; doi:10.1371/journal.ppat.1003758)
Supplement: Figure S1 — Growth rate of ancestors exposed to different concentrations of kanamycin. Growth rates were estimated at different concentrations of kanamycin for the four ancestors in a 96-well plate using an automated plate reader (n = 3). OD600 in CTM pH 7.8 was measured every 5 minutes for 24 hours at 37°C with continuous shaking. Raw OD values were normalised to a blank well and Ln transformed before analysis. Growth rates declined with increasing concentration of kanamycin (Figure S1). (DOCX) [file ppat.1003758.s001.docx]

**Figure S1- growth rate of ancestors exposed to different concentrations of kanamycin**

Growth rates were estimated at different concentrations of kanamycin for the four ancestors in a 96-well plate using an automated plate reader (n=3). OD_600_ in CTM pH 7.8 was measured every 5 minutes for 24 hours at 37°C with continuous shaking. Raw OD values were normalised to a blank well and Ln transformed before analysis.

Fig S1. Mean growth rates of the ancestral strains while exposed to different concentrations of kanamycin. Dark greys are non-competent ancestors and light/white are the competent ancestors. Error bars are SE of the mean.
