# Supplementary material for: Conservative Sex and the Benefits of Transformation in Streptococcus pneumoniae
Source: PLoS Pathog. 2013 Nov 14;9(11):e1003758. doi: 10.1371/journal.ppat.1003758 (PMC3828180; doi:10.1371/journal.ppat.1003758)
Supplement: Figure S2 — Growth rates of the four ancestors. Growth rates were estimated within a 96-well plate using an automated plate reader (n = 5). Cultures were started with 3*105 cells in 200 µL of CTM pH 7.8. The OD600 was measured every 5 minutes for 24 hours at 37°C with continuous shaking. Raw OD values were normalised to a blank well and Ln transformed before analysis. Then the steepest slope over a 35 minute period was determined. The growth rates were analysed with a one-way ANOVA using R (Figure S2). (DOCX) [file ppat.1003758.s002.docx]

**Figure S2 – Growth rates of the four ancestors**

Growth rates were estimated within a 96-well plate using an automated plate reader (n=5). Cultures were started with 3*10^5^ cells in 200µL of CTM pH 7.8. The OD_600_ was measured every 5 minutes for 24 hours at 37°C with continuous shaking. Raw OD values were normalised to a blank well and Ln transformed before analysis. Then the steepest slope over a 35 minute period was determined. The growth rates were analysed with a one-way ANOVA using R (Fig S2).

Figure S2 Mean growth rates of ancestral strains. No significant difference was detected between the strains (one-way ANOVA: F_3,16_ = 0.8637; p = 0.4801). Error bars are SE of the mean.
