# Supplementary material for: Conservative Sex and the Benefits of Transformation in Streptococcus pneumoniae
Source: PLoS Pathog. 2013 Nov 14;9(11):e1003758. doi: 10.1371/journal.ppat.1003758 (PMC3828180; doi:10.1371/journal.ppat.1003758)
Supplement: Figure S3 — Natural transformation in the chemostat environment. Populations containing mixes of reciprocally marked (rifampicin or streptomycin resistance) ancestors were tested for the frequency of cells with both markers to test for natural transformation in the chemostat environment. Double marked cells that were found in chemostats containing a mix of the non-competent FP5 ancestor would be the result of mutation, while those found in the chemostats with a mix of the competent Rx1 ancestor would be the result of both mutation and recombination. If the rate at which double mutants occur in the mix of Rx1 is higher than the mix of FP5 then this excess will be the result of transformation. To test this, chemostats containing ¼ CTM pH 7.8 were inoculated with either a 1∶1 mix (n = 3 for each mix) of streptomycin and rifampicin resistant FP5 (non-competent) or Rx1 (competent). After 24 hours of growth at 37°C, the population density and the number of double marked cells was determined on blood agar plates, which were supplemented with 100 µg/mL streptomycin and 4 µg/mL rifampicin where necessary. Since the population sizes were similar after 24 hours, the number of double marked cells in the total population was compared between ancestor types in R using a GLM model with a Poisson distribution (Figure S3). (DOCX) [file ppat.1003758.s003.docx]

**Figure S3 – Natural transformation in the chemostat environment**

Populations containing mixes of reciprocally marked (rifampicin or streptomycin resistance) ancestors were tested for the frequency of cells with both markers to test for natural transformation in the chemostat environment. Double marked cells that were found in chemostats containing a mix of the non-competent FP5 ancestor would be the result of mutation, while those found in the chemostats with a mix of the competent Rx1 ancestor would be the result of both mutation and recombination. If the rate at which double mutants occur in the mix of Rx1 is higher than the mix of FP5 then this excess will be the result of transformation. To test this, chemostats containing ¼ CTM pH 7.8 were inoculated with either a 1:1 mix (n=3 for each mix) of streptomycin and rifampicin resistant FP5 (non-competent) or Rx1 (competent). After 24 hours of growth at 37°C, the population density and the number of double marked cells was determined on blood agar plates, which were supplemented with 100µg/mL streptomycin and 4µg/mL rifampicin where necessary. Since the population sizes were similar after 24 hours, the number of double marked cells in the total population was compared between ancestor types in R using a GLM model with a Poisson distribution (Fig S3).

Figure S3. Distribution of double marked cells by ancestor type. Double marked cells appeared more often in chemostats with competent populations than in chemostats with non-competent populations (GLM with Poisson distribution: χ^2^=272.37, df = 1, p < 0.0001). This shows that transformation occurs more often than mutation in the chemostat environment.
