# Supplementary material for: Conservative Sex and the Benefits of Transformation in Streptococcus pneumoniae
Source: PLoS Pathog. 2013 Nov 14;9(11):e1003758. doi: 10.1371/journal.ppat.1003758 (PMC3828180; doi:10.1371/journal.ppat.1003758)
Supplement: Table S1 — Table of all mutations across DNA repair genes for all evolved populations. (DOCX) [file ppat.1003758.s004.docx]

**Table S1 – Table of all mutations across DNA repair genes for all evolved populations**

Positions of amino acid changes in DNA repair genes within the evolved populations. Evolved populations are organised by treatment and genes are organised by repair function. For the analysis described in the manuscript, genes with more than one mutation in a particular evolved line were conservatively scored as having one mutation (i.e. presence of mutation) rather than the absolute number of mutations.

|  |  |  | **No competence** | | | | | | | | **Competence** | | | | | | | |
| --- | --- | --- | --- | --- | --- | --- | --- | --- | --- | --- | --- | --- | --- | --- | --- | --- | --- | --- |
|  |  |  | **No stress** | | | | **Stress** | | | | **No stress** | | | | **Stress** | | | |
| **Pathway** | **Gene #^1^** | **Gene** | 1 | 2 | 3 | 4 | 1 | 2 | 3 | 4 | 1 | 2 | 3 | 4 | 1 | 2 | 3 | 4 |
| Base Excision Repair | spr1055 | ung |  |  |  |  |  |  | V52A |  |  |  |  |  |  |  |  |  |
| Base Excision Repair | spr0872 | mutM |  |  |  |  |  |  | P93L |  |  |  |  |  |  |  |  |  |
| Base Excision Repair | spr1108 | mutY |  |  |  |  |  |  |  |  |  |  |  |  |  |  |  | M53R |
| Base Excision Repair | spr0537 | recJ |  | T404I |  |  |  |  |  |  |  |  |  |  |  |  |  |  |
|  | spr1024 | ligA |  |  |  |  |  |  | V212I |  |  |  |  |  |  |  |  |  |
| Nucleotide excision repair |  | uvrB |  |  |  |  |  |  | S122N; G321E |  |  |  |  |  |  |  |  |  |
|  | spr1118 |  |  |  |  |  |  |  |  |  |  |  |  |  |  |  |  |  |
| Nucleotide excision repair | spr0543 | uvrC |  |  |  |  |  |  |  |  |  |  |  |  |  |  |  | H616Y |
| Nucleotide excision repair | spr0215 | rpoA |  |  |  |  |  |  |  |  |  |  |  | T301I | M263I |  | E332K |  |
| Nucleotide excision repair |  | rpoB | R168C; R1010H |  | R864C |  |  |  | G559S | T726A |  | V1094I |  |  |  | T723A |  |  |
|  | spr1777 |  |  |  |  |  |  |  |  |  |  |  |  |  |  |  |  |  |
| Nucleotide excision repair | spr1776 | rpoC |  | E1249K |  |  |  |  |  |  |  |  |  | D237N |  |  |  |  |
| Nucleotide excision repair |  | mfd | +t 1256 |  |  | M407T |  |  | D12N; Q985R | S229L |  |  |  |  |  |  |  |  |
|  | spr0006 |  |  |  |  |  |  |  |  |  |  |  |  |  |  |  |  |  |
| Mismatch excision repair |  | mutL^2^ |  |  |  | P154L; R371stop |  |  | A131V |  |  |  |  |  |  |  |  |  |
|  | spr0160 |  |  |  |  |  |  |  |  |  |  |  |  |  |  |  |  |  |
| Mismatch excision repair | spr0795 | dnaE |  |  |  |  |  |  | Q291R |  |  |  |  |  |  |  |  |  |
| Mismatch excision repair |  | polC | M275V | M1363T |  | V1168I | D1285Y |  | H201R; A576D |  |  |  |  |  |  |  |  |  |
|  | spr0251 |  |  |  |  |  |  |  |  |  |  |  |  |  |  |  |  |  |
| Mismatch excision repair | spr0896 | dnaQ |  | S191L |  |  |  |  |  |  |  |  |  |  |  |  |  |  |
| Mismatch excision repair | spr0769 | dnaX |  | A266T |  |  |  |  |  |  |  |  |  |  |  |  |  |  |
| Homologous recombination | spr1581 | priA |  |  |  |  |  |  | T806M |  |  |  |  |  |  |  |  |  |
| Homologous recombination | spr2032 | recF |  |  |  | L381P |  |  |  |  |  |  |  |  |  |  |  |  |
| Homologous recombination |  | recG |  |  |  |  |  |  | K133E; A487V |  |  |  |  |  |  |  |  |  |
|  | spr1539 |  |  |  |  |  |  |  |  |  |  |  |  |  |  |  |  |  |
| Homologous recombination | spr1084 | recN |  |  |  |  |  |  | E398K |  |  |  |  |  |  |  |  |  |
| Non-homologous end-joining |  | gyrA |  |  |  |  |  |  | P58S; T781I |  |  |  |  |  |  |  |  |  |
|  | spr1099 |  |  |  |  |  |  |  |  |  |  |  |  |  |  |  |  |  |
| Non-homologous end-joining |  | topA |  |  |  | H505L | V129I; F634S |  |  |  |  |  |  |  |  |  |  | G461V |
|  | spr1141 |  |  |  |  |  |  |  |  |  |  |  |  |  |  |  |  |  |
| Modulation of nucleotide pools | spr0023 | dut |  |  |  |  |  |  | R125C |  |  |  |  |  |  |  |  |  |
| Modulation of nucleotide pools |  | nrdE |  |  | L537I |  | T110I |  | P241L; M526T |  |  |  |  |  |  |  | T714I | T678I |
|  | spr1065 |  |  |  |  |  |  |  |  |  |  |  |  |  |  |  |  |  |

^1^ Gene # based on *S. pneumoniae* R6 genome annotation at http://www.streppneumoniae.com/ and GenBank accession number AE007317.

^2^ mutL (Kegg database annotation) is named hexB in GenBank.
