# Supplementary material for: Conservative Sex and the Benefits of Transformation in Streptococcus pneumoniae
Source: PLoS Pathog. 2013 Nov 14;9(11):e1003758. doi: 10.1371/journal.ppat.1003758 (PMC3828180; doi:10.1371/journal.ppat.1003758)
Supplement: Table S2 — Mutation rates calculated from mutations in coding sites and synonymous sites. (DOCX) [file ppat.1003758.s005.docx]

**Table S2 – Mutation rates calculated from mutations in coding sites and synonymous sites**

Table S2: Mutation rates for each population based on synonymous site changes, coding site changes and total genomic changes.

| treatment | | | source of calculations | | |
| --- | --- | --- | --- | --- | --- |
| competence | kanamycin | population | synonymous | coding region | total genome |
| no | no | 1 | 4.97E-08 | 4.55E-08 | 4.27E-08 |
| no | no | 2 | 7.32E-08 | 7.29E-08 | 7.01E-08 |
| no | no | 3 | 3.66E-08 | 3.70E-08 | 3.88E-08 |
| no | no | 4 | 1.39E-07 | 1.13E-07 | 1.06E-07 |
| no | yes | 5 | 3.66E-08 | 3.19E-08 | 3.29E-08 |
| no | yes | 6 | 3.14E-08 | 2.56E-08 | 2.40E-08 |
| no | yes | 7 | 4.08E-07 | 2.99E-07 | 2.91E-07 |
| no | yes | 8 | 3.14E-08 | 4.38E-08 | 4.17E-08 |
| yes | no | 9 | 3.66E-08 | 3.87E-08 | 4.07E-08 |
| yes | no | 10 | 3.66E-08 | 2.79E-08 | 3.09E-08 |
| yes | no | 11 | 2.88E-08 | 1.88E-08 | 2.06E-08 |
| yes | no | 12 | 2.88E-08 | 3.30E-08 | 3.43E-08 |
| yes | yes | 13 | 4.44E-08 | 4.90E-08 | 4.81E-08 |
| yes | yes | 14 | 2.35E-08 | 4.73E-08 | 4.56E-08 |
| yes | yes | 15 | 5.49E-08 | 4.21E-08 | 3.97E-08 |
| yes | yes | 16 | 4.18E-08 | 4.38E-08 | 4.76E-08 |
